# Isolated fractures of the greater tuberosity of the proximal humerus: A long-term retrospective study of 30 patients

**DOI:** 10.3109/17453674.2011.618912

**Published:** 2011-11-25

**Authors:** Stefan G Mattyasovszky, Klaus J Burkhart, Christopher Ahlers, Dirk Proschek, Sven-Oliver Dietz, Inma Becker, Stephan Müller-Haberstock, Lars P Müller, Pol M Rommens

**Affiliations:** ^1^Department of Trauma Surgery, Center for Musculoskeletal Surgery; ^2^Department of Radiology, University Medical Center, Johannes Gutenberg University Mainz, Germany

## Abstract

**Background and purpose:**

The diagnosis and treatment of isolated greater tuberosity fractures of the proximal humerus is not clear-cut. We retrospectively assessed the clinical and radiographic outcome of isolated greater tuberosity fractures.

**Patients and methods:**

30 patients (mean age 58 (26–85) years, 19 women) with 30 closed isolated greater tuberosity fractures were reassessed after an average follow-up time of 3 years with DASH score and Constant score. Radiographic outcome was assessed on standard plain radiographs.

**Results:**

14 of 17 patients with undisplaced or slightly displaced fractures (≤ 5 mm) were treated nonoperatively and had good clinical outcome (mean DASH score of 13, mean Constant score of 71). 8 patients with moderately displaced fractures (6–10 mm) were either treated nonoperatively (n = 4) or operatively (n = 4), with good functional results (mean DASH score of 10, mean Constant score of 72). 5 patients with major displaced fractures (> 10 mm) were all operated with good clinical results (mean DASH score of 14, mean Constant score of 69). The most common discomfort at the follow-up was an impingement syndrome of the shoulder, which occurred in both nonoperatively treated patients (n = 3) and operatively treated patients (n = 4). Only 1 nonoperatively treated patient developed a non-union. By radiography, all other fractures healed.

**Interpretation:**

We found that minor to moderately displaced greater tuberosity fractures may be treated successfully without surgery.

Isolated fractures of the greater tuberosity account for approximately 20% of all proximal humeral fractures (Chun et al. 1994, Kim et al. 2005, Gruson et al. 2008). They are often associated with anterior glenohumeral dislocation or can result from an impaction injury, also called a shear injury, against the lower surface of the acromion or superior glenoid (Court-Brown et al. 2001, George 2007).

The diagnosis and classification of isolated greater tuberosity fractures are mainly based on standard plain radiographs. However, these fractures may be challenging to identify because of osseous overlap; Ogawa et al. (2003) reported that two-thirds of these fractures were missed on initial evaluation. It is generally accepted that undisplaced and slightly displaced (≤ 5 mm) fractures of the greater tuberosity should be treated non-surgically, but the magnitude of displacement that warrants surgical intervention is debatable (Park et al. 1997, Gaebler et al. 2003, Platzer et al. 2005).

Although isolated greater tuberosity fractures are well recognized and frequently described to be a special group of proximal humeral fractures, only a few studies have specifically evaluated the clinical outcome of these injuries. We retrospectively assessed the clinical outcome of isolated greater tuberosity fractures.

## Patients and methods

78 patients with isolated fractures of the greater tuberosity of the humerus were seen at our trauma surgery department over a 10-year period (1995–2005). 30 patients (19 women) with a mean age of 58 (26–85) years with 30 isolated greater tuberosity fractures of the proximal humerus could be re-examined a mean of 3 (0.7–10) years after treatment. The remaining 48 patients were lost for the following reasons: 6 patients had died, 21 patients denied re-evaluation either because of good clinical result or because they were unable to attend, and 21 patients had moved and could not be reached.

The mechanisms of injury were either falling from a height or falling on stairs (n = 10), vehicle trauma or motorcycle accidents (n = 8), recreational accidents (n = 8), or sporting trauma (n = 4). There was no open fracture. The right proximal humerus had been affected more often (n = 17) than the left (n = 13).

### Clinical evaluation

All patients were evaluated using an interview with a detailed questionnaire regarding the patient's general health. The radiographic findings, the documented operative reports, and all the charts were reviewed. General function of the shoulder and rotator cuff function was determined clinically by standard tests, with measurements of motion using a goniometer. Impingement syndrome was diagnosed by history and physical exam using Neer's clinical sign. For the objective assessment, the DASH score (Hudak et al. 1996) and the Constant score (CS) (Constant and Murley 1987) were used. Shoulder strength was assessed according to the recommended methodology for the CS. DASH score was graded as excellent (0–24), good (25–49), moderate (50–74), or poor (75–100). The CS results were given in the categories excellent (86–100), good (71–85), moderate (56–70), and poor (0–55).

### Radiographic evaluation

Primary standard plain radiographs with true glenoid anteroposterior (AP) and trans-scapular lateral view (Y-view) of the shoulder were retrospectively evaluated by two independent examiners (a shoulder specialist and a senior radiologist). If available, complementary to standard plain radiographs (axillary views), CT scans, or MRI scans were interpreted. The extent of fragment dislocation was measured in mediolateral and craniocaudal direction. In addition, we evaluated the number of fragments. Measurements on radiographic images were performed using a conventional millimeter scale if conventional film radiographs were available, but most of the radiographs were available in digital form. For evaluation of these images, we used the DICOM viewing software ConVis (Systema GmbH, Koblenz, Germany) for viewing, for digital measurements, and for distance calculations. If digital CT or MRI images were available, measurements were performed in reconstructed coronal and sagittal planes (corresponding to true AP and lateral view on radiographs) using the 3D MPR module of AquariusNet software (TeraRecon, San Mateo, CA). In cases where both radiographic and cross-sectional imaging was available, measurements of cross-sectional modality were given preference under the assumption of higher accuracy. The degree of fragment dislocation was classified (undisplaced to minor: ≤ 5 mm; moderate: 6–10 mm; major: > 10 mm) and calculated as the distance between the upper surface of the humeral head and the upper margin of the displaced main fragment, or the distance between the outer surface of the humeral head and the outer margin of the displaced main fragment, respectively. Anterior and cranial displacements are given as positive values, and posterior and caudal displacements as negative values.

The follow-up images were assessed for radiographic healing, whereas the presence of posttraumatic heterotopic ossification (HO) was classified into three categories (0 = normal, 1 = moderate, and 2 = severe). In accordance with the Kellgren-Lawrence classification, osteoarthritis (OA) of the glenohumeral joint was classified into 5 grades (0 = normal, 1 = questionable, 2 = incipient or mild OA (slight narrowing of the joint space), 3 = moderate OA (distinct narrowing of the joint, bone cysts, and sclerosis), and 4 = severe OA (severe structural disorder of the joint)).

### Statistics

We used analysis of variance (ANOVA) to compare the clinical outcomes of the 3 groups. 95% CIs were calculated. We used SPSS version 10.07 software.

## Results

### Epidemiology and injury morphology

**Figure 1.**
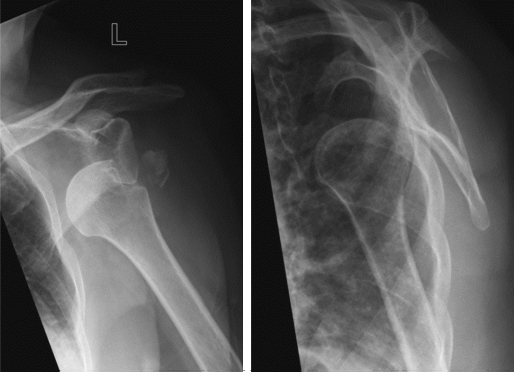
The left shoulder of a 64-year-old patient (no. 3, Table 3), who suffered an anterior glenohumeral dislocation of the left shoulder with an avulsed greater tuberosity.

One third of the injuries (n = 10) occurred at 50–59 years of age; 20 patients were 50 years of age or older. Only 7 patients were younger than 39 years of age. 9 patients also had a glenohumeral dislocation, which was anterior in all cases (Figure 1). They were primarily treated with closed reduction and immobilization with a Gilchrist bandage. 4 of these patients had a surgical intervention later on.

A Hill-Sachs lesion as an impacted area of the posterosuperior aspect of the humeral head was diagnosed in 4 patients with shoulder dislocation. A rotator cuff lesion (in 2 patients) and a Bankart's lesion (in 1 patient) were diagnosed in MRI examinations. 10 patients suffered either an additional craniocerebral trauma (n = 4) or injuries of the extremities (n = 6), whereas 3 patients had multiple injuries. 19 patients had no other lesions.

18 patients were treated nonoperatively with immobilization using a Gilchrist bandage for 7–10 days until pain relief was achieved, followed by oscillating movements of the arm. After 3–4 weeks, active motion was started. Patients were allowed to bear weight on their arms after 6–8 weeks.

**Figure 2.**
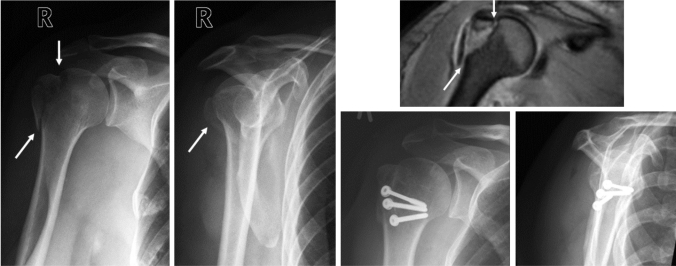
Patient no. 9 (Table 3) before and after open reduction and internal fixation with cannulated screws of a moderately displaced fracture of the greater tuberosity (white arrows). An MRI scan illustrates the fracture line (white arrows).

**Figure 3.**
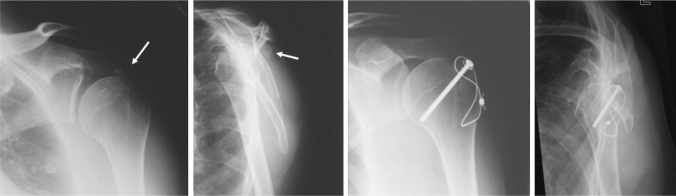
Patient no. 6 (Table 3) before and after open reduction and internal fixation of greater tuberosity fragments with major displacement (white arrows). The multiple fragments were fixed with a screw and a tension wire.

**Figure 4.**
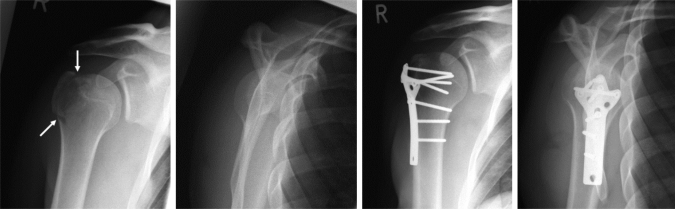
Patient no. 18 (Table 3) before and after open reduction and plate fixation of a moderately displaced fracture of the greater tuberosity (white arrows).

The 12 operated patients were treated by open reduction and internal fixation (ORIF). The fixation techniques varied. If the avulsed fragment was well reduced but unstable after closed reduction of the shoulder dislocation and/or several fragments were present, a minimally invasive fixation through a mini-open transdeltoidal approach was used (n = 3). Percutaneous fixation of the fragment(s) with temporary K-wire(s) was performed and cannulated screws were placed over the temporary K-wires. If closed reduction failed (n = 9), open reduction using a transdeltoid lateral approach was performed. Internal fixation with 1–3 screws (Figure 2 and Table 3) was the most common method used (n = 9) used. In 1 case, a combination of a screw with a tension wire (Figure 3 and Table 3) was necessary for adequate stabilization, and 2 fractures were fixed with a plate (Figure 4 and Table 3). As comminuted tuberosity fractures are essentially rotator cuff tears with an attached bony fragment, appropriate sutures were used as part of the fixation technique (n = 4). All wounds healed without complications.

### Clinical outcome

The 17 patients with an undisplaced or slightly displaced fracture (≤ 5mm) had good to excellent clinical results (Table 1). 14 of these fractures were treated without surgery and 3 patients were operated. 1 of these 3 patients (no. 20, Table 3) had an excellent clinical outcome whereas the 2 other patients had poor clinical outcomes (patients 3 and 4) (Table 3). The 8 patients with moderately displaced fractures (6–10 mm) were either treated nonoperatively (n = 4) or operatively, with good to excellent clinical outcome (Table 1).

**Table 1.** Overview of clinical evaluation considering fragment displacement

Scores	Degree of fragment displacement **^a^**			p-value
	None/minor (n = 17)	Moderate (n = 8)	Major (n = 5)	
DASH score **^b^**				
75–100 (poor)	–	–	–	
50–74 (moderate)	1	–	1	
25–49 (good)	3	1	–	
< 25 (excellent)	13	7	4	
Mean (SD)	13 (17)	10 (12)	14 (24)	0.9
95% CI	4 to 22	0 to 20	–16 to 44	
Constant score				
86–100 (excellent)	3	1	–	
71–85 (good)	7	5	3	
56–70 (moderate)	3	2	1	
< 55 (poor)	4	–	1	
Mean (SD)	71 (18)	72 (16)	69 (17)	1.0
95% CI	62 to 80	59 to 85	48 to 90	

**^a^** None/minor indicates displacement of ≤ 5 mm, moderate indicates displacement of 6–10 mm, and major indicates displacement of > 10 mm.

**^b^** DASH: Disabilities of the Arm, Shoulder and Hand scale.

All 5 patients with major displaced fractures (> 10 mm) were operated and 4 had good to excellent results (Table 1), although 1 patient (no. 27, Table 3) who was treated with open fixation using 3 screws and suture fixation technique had impingement symptom at the follow-up and had a moderate DASH score of 57 points with a poor CS of 41 points.

No patients had poor results in the DASH score, whereas interestingly 4 patients with minor displacement and one patient with major displacement had poor results as evaluated with the CS (Table 3). In summary, according to the degree of fragment displacement, there was no statistically significant difference in clinical outcome considering the mean DASH score and CS between the 3 groups (Table 1). Irrespective of the treatment strategy and the initial degree of fragment displacement, 7 patients complained about impingement symptoms at the follow-up. 3 of these patients with no displacement or minor displacement had been treated nonoperatively and 4 had been treated operatively, with 2 patients having minor displacement, 1 patient moderate, and 1 patient a major displacement. At the time of the follow-up, there was no statistically significant difference between the 3 groups regarding active ROM of the affected shoulder (Table 2).

**Table 2.** Active ROM at the time of follow-up examination

	Degree of fragment displacement and ROM **^a^**			p-value
	None/minor	Moderate	Major	
	(n = 17)	(n = 8)	(n = 5)	
Forward flexion	155 (105–170)	149 (100–170)	142 (130–155)	0.6
Abduction	147 (90–170)	142 (95–175)	146 (130–160)	0.9
External rotation	54 (30–70)	49 (30–70)	40 (20–60)	0.2
Internal rotation	72 (50–90)	69 (40–90)	68 (50–80)	0.8

**^a^** The range of motion (ROM) of the affected arm is presented as the mean value in degrees with the range in parentheses.

**Table 3.** Synopsis of clinical and radiographic evaluation of patients with isolated greater tuberosity fractures of the humerus

Clinical evaluation							Radiographic evaluation						
A	B	C	D	E	F	G	H	I	J	K	L	M	N
*No/minor displacement (≤ 5 mm)*													
3	F	64	3 screws+ suture	impingement	4.2	50	anterior	+, CT	2	4	0	2	2
4	F	33	–	impingement	8.3	79	–	+	1	0	0	0	0
5	M	26	–	–	2.5	88	anterior	+	1	4	0	0	0
7	F	58	–	–	10	64	–	+	2	0	–5	0	0
10	M	54	–	–	4.2	81	–	+	1	0	0	0	0
11	F	71	–	–	36	70	anterior	+	1	0	0	0	2
12	F	38	–	–	0	82	–	+	2	0	0	0	0
13	M	57	–	–	7.5	83	–	+	1	0	0	0	0
14	M	40	–	–	0	89	–	+, CT	3	–4	4	0	0
15	F	74	–	–	32	42	–	+, MRI	1	0	5	0	0
16	M	65	–	impingement	58	38	anterior	+	3	5	0	2	0
20	M	53	2 screws	–	0	92	–	+	1	4	0	0	0
22	F	80	–	–	8.3	80	–	+	1	0	5	1	0
23	F	65	–	–	0	80	anterior	+	3	0	0	0	0
24	F	36	2 screws	impingement	34.2	44	–	+	1	0	0	0	0
28	F	37	–	impingement	6.7	57	–	+, CT	2	4	0	0	0
29	F	58	–	non-union	11.7	81	–	+, MRI	1	0	0	0	0
													
*Moderate displacement (6–10 mm)*													
1	F	83	–	–	23	51	anterior	+	3	6	5	0	2
2	F	85	–	–	7.5	78	–	+	2	0	–10	0	3
8	M	48	3 screws	–	1.7	83	–	+, CT	3	0	6	0	0
9	F	53	3 screws+suture	impingement	33	44	anterior	+, MRI	2	0	6	0	0
17	F	53	–	–	3.3	80	–	+, CT	1	0	6	0	0
18	M	31	plate	–	2.5	89	anterior	+	3	9	5	1	0
21	M	76	–	–	5	77	–	+	1	6	8	0	2
26	F	68	3 screws	–	5.8	74	anterior	+	2	0	7	0	0
													
*Major displacement (> 10 mm)*													
6	F	80	1 screw + TW	–	1.7	77	–	+	> 3	10	–20	2	2
19	M	55	plate+suture	–	0	78	–	+, CT	1	0	–17	2	
25	F	68	2 screws	–	13	64	–	+	2	11	7	0	2
27	F	60	3 screws+suture	impingement	57	41	–	+	1	7	12	0	2
30	M	63	2 screws	–	0	85	–	+	1	30	6	2	3

A Pat no B Sex C Age D ORIF E Discomfort shoulder impingement syndrome was clinically determined with the Neer sign F DASH score G Constant score H Shoulder dislocation I Imaging +: radiography CT: computed tomography MRI: magnetic resonance imaging J No. of fragments Fragment displacement (anterior and cranial displacement are given as positive values, posterior and caudal as negative values) K Vertical L Horizontal M Heterotopic ossification 0 = none 1 = moderate 2 = severe N Osteoarthritis – Kellgren-Lawrence classification 0 = normal 1 = questionable 2 = incipient or mild 3 = moderate 4 = severe

### Radiographic outcome

Radiographs on the day of injury showed that 15 patients had a greater tuberosity fracture consisting of 1 fragment, whereas in 15 patients the fractured greater tuberosity consisted of 2 or more fragments (Table 3). 17 patients had greater tuberosity fractures with no or only minor displacement (Table 3). 8 patients had moderate displacement and 5 patients had major displacement. 6 patients had an additional CT for better evaluation of the lesion. We could not find any effect on treatment strategy of the findings of these additional examinations.

**Figure 5.**
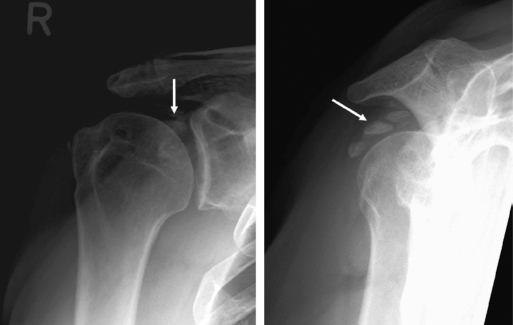
Radiographs of the right shoulder illustrating severe periarticular calcification (white arrows) of a patient (no. 30, Table 3) who was treated operatively with ORIF with screws for a major displaced fracture of the greater tuberosity. After radiographic healing, the metal was removed.

All lesions healed without a measurable loss of reduction. 1 patient (no. 29, Table 3) with an undisplaced fracture that was treated nonoperatively developed a non-union. 9 patients, 5 of which were treated operatively and 4 of which were treated nonoperatively, showed moderate to severe signs of OA of the shoulder. 2 patients who developed OA had no displacement or minor displacement of the fracture, 3 patients had moderate displacement, and 4 had major displacement. Furthermore, 7 patients had moderate (n = 2) to severe (n = 5) heterotopic ossification of the shoulder that mostly occurred at the insertion site of the supraspinatus tendon (Figure 5). 5 of these patients, 1 with moderate ossifications and 4 with severe ossifications, were treated operatively.

## Discussion

The amount of fragment displacement of isolated greater tuberosity fractures that warrants surgical intervention has been discussed since the early 1970s (Neer 2006). Posterosuperior displacement of the greater tuberosity of more than 5 mm from the anatomic position can result in malunion and impingement of the shoulder due to an altered rotator cuff insertion site influencing the motion in the glenohumeral joint (Bono et al. 2001). The recommendation of Neer (2006) to treat displacements of the tuberosity of less than 1 cm nonoperatively has been revised, and in the current literature it is recommended that surgical fixation be used for fractures with more than 5 mm of displacement in the general population or more than 3 mm of displacement in active patients with frequent overhead activity (Park et al. 1997, George 2007).

Two surgical approaches have been described for open reduction and internal fixation of proximal humeral fractures: a deltopectoral and a transdeltoid lateral approach. In the present study, the transdeltoid lateral approach was used for all patients treated surgically. This approach is commonly used for open treatment of injuries of the rotator cuff. Whereas screw fixation is the rule, additional fixation techniques might be accurate in specific cases. If the fracture consists of one fragment in patients with good bone quality, screw fixation is the procedure of choice. It may be enhanced by a tension banding wiring of the rotator cuff tendons. In displaced and comminuted fractures, the fracture fragments are held together by the soft tissues and still attached to the humerus by the periosteum. Osteosutures with large-caliber resorbable sutures or nonresorbable surgical tape, and tension band wiring provide adequate relative stability. The concomitant use of arthroscopic techniques was only recently discussed in the literature (Ji et al. 2007, 2010, Song and Williams. 2008). With recent advancements in shoulder arthroscopy, minimally displaced greater tuberosity fractures and associated rotator cuff lesions can be treated successfully arthroscopically (Ji et al. 2007, 2010, Song and Williams 2008). Limitations of arthroscopic repair are insufficient vision, relevant displacement, or fixed retraction of the avulsed fragment. In our study, none of the patients were treated arthroscopically.

According to our findings, it is not indicated to surgically reduce displacements of 3–5 mm as suggested in the literature (McLaughlin 1963, Paavolainen et al. 1983, Park et al. 1997, Kim et al. 2005, George 2007), as most of the patients in our study with minor displacement of the fracture (≤ 5 mm) and half of the patients with moderate displacement (6–10 mm) were treated nonoperatively and achieved good to excellent clinical results. Although 4 of the 5 patients with dislocations of more than 1 cm were treated surgically with a good clinical outcome, we cannot conclude that it was neccessary to operate these patients.

There are drawbacks related to operative treatment. Heterotopic ossification and appearance of OA was more common in operatively treated patients (Table 3). Randomized clinical trials are needed to identify the best treatment of these fractures.
